# Approximate Series Solution of Nonlinear Singular Boundary Value Problems Arising in Physiology

**DOI:** 10.1155/2014/945872

**Published:** 2014-02-20

**Authors:** Randhir Singh, Jitendra Kumar, Gnaneshwar Nelakanti

**Affiliations:** Department of Mathematics, Indian Institute of Technology Kharagpur, Kharagpur 721302, India

## Abstract

We introduce an efficient recursive scheme based on Adomian decomposition method (ADM) for solving nonlinear singular boundary value problems. This approach is based on a modification of the ADM; here we use all the boundary conditions to derive an integral equation before establishing the recursive scheme for the solution components. In fact, we develop the recursive scheme without any undetermined coefficients while computing the solution components. Unlike the classical ADM, the proposed method avoids solving a sequence of nonlinear algebraic or transcendental equations for the undetermined coefficients. The approximate solution is obtained in the form of series with easily calculable components. The uniqueness of the solution is discussed. The convergence and error analysis of the proposed method are also established. The accuracy and reliability of the proposed method are examined by four numerical examples.

## 1. Introduction

In this paper we propose an efficient recursive scheme based on Adomian decomposition method (ADM) for solving a class of nonlinear singular boundary value problems (SBVPs) that arising in physiology [1–5]:

(1) $$\begin{array}{c} {\left(x^{\alpha }y^{\prime }\left(x\right)\right)}^{\prime }=x^{\alpha }f\left(x,y\left(x\right)\right),\;0<x<1,\;\;\alpha \geq 0,\\ y^{\prime }\left(0\right)=0,\;\;ay\left(1\right)+by^{\prime }\left(1\right)=c. \end{array}$$

Here *a* > 0, *b* ≥ 0, and *c* are any finite real constants. We assume that for (*x*, *y*)∈{(0,1) × ℝ}, the functions *f*(*x*, *y*) and ∂*f*/∂*y* are continuous and ∂*f*/∂*y* ≥ 0. Singular boundary value problems frequently arise in the modeling of many problems in biological, physical, and engineering sciences. For example, it arises in the study of steady-state oxygen diffusion in a spherical cell with Michaelis-Menten uptake kinetics [6] with *α* = 2 and *f*(*x*, *y*) = *N*_1_ *y*/*y* + *K*_1_, where *N*_1_ and *K*_1_ are positive real constants. In heat conduction model in human head [7, 8] with *α* = 2, *f*(*x*, *y*) = −*a*_0_ *e* ^−*b*_0_*y*^, where *a*_0_ and *b*_0_ are positive reals.

There is considerable literature on the numerical treatment of singular boundary value problems [1–11] and many of the references therein. The main difficulty of the problem (1) is that the singularity behavior occurs at *x* = 0. Various efficient numerical techniques have been used to deal with such SBVPs, such as finite difference method (FDM) [9], cubic spline method (CSM) [1], and B-spline method (BSM) [2]. Although, these techniques are efficient and have many advantages, a huge amount of computational work is needed which combines some root-finding methods to obtain accurate numerical solution especially for nonlinear SBVPs. Recently, some newly developed approximate methods were also applied to deal with such SBVPs, such as variational iteration method (VIM) [5, 11], Adomian decomposition method (ADM) [3], and modified Adomian decomposition method (MADM) [4]. It is well known that solving nonlinear two-point boundary value problems (BVPs) using ADM/MADM is always a computationally involved task. Since it requires computation of unknown constants in a sequence of nonlinear or more difficult transcendental equations. Moreover, in some cases these unknown constants may not be uniquely determined and this may be the major disadvantage of ADM/MADM for solving nonlinear BVPs.

The aim of this work is to introduce an efficient recursive scheme to overcome the difficulties that occur in the ADM or MADM for solving nonlinear SBVPs (1). This approach is based on a modification of the ADM; here we use all the boundary conditions to derive an integral equation before establishing the recursive scheme for the solution components. In fact, we develop the recursive scheme without any undetermined coefficients while computing the solution components. Unlike the ADM, the proposed scheme avoids solving a sequence of nonlinear algebraic or transcendental equations for the undetermined coefficients. The approximations of the solution are obtained in the form of series with easily computable components. The sufficient condition that guarantees the existence of a unique solution of the problem (1) is proved. The convergence analysis and the error estimation are also discussed. Finally, the accuracy and reliability of the proposed method are examined by four numerical examples.

### 1.1. The Review of ADM

In this subsection, we briefly describe the ADM for solving SBVPs (1). Recently, many researchers [3, 4, 12–23] have applied the ADM to deal with many different scientific models. According to the ADM we rewrite (1) in a operator form as

(2) $$\begin{array}{c} Ly\left(x\right)=f\left(x,y\left(x\right)\right), \end{array}$$

where *L* = *x* ^−*α*^ *d*/*dx*(*x* ^*α*^ *d*/*dx*) is a second-order linear differential operator. In [24] Wazwaz defines the inverse operator *L* ^−1^ as

(3) $$\begin{array}{c} L^{-1}\left[\cdot \right]=\overset{x}{\underset{0}{\int }}x^{-\alpha }\overset{x}{\underset{0}{\int }}x^{\alpha }\left[\cdot \right]dx\;dx. \end{array}$$

Operating *L* ^−1^[·] on both sides of (2) and using the condition *y*′(0) = 0, we have

(4) $$\begin{array}{c} y\left(x\right)=y\left(0\right)+L^{-1}\left[f\left(x,y\left(x\right)\right)\right]. \end{array}$$

The main idea of the ADM depends on decomposing the solution *y*(*x*) and the nonlinear function *f*(*x*, *y*(*x*)) by an infinite series as

(5) $$\begin{array}{c} y\left(x\right)=\overset{\infty }{\underset{j=0}{\sum }}y_{j}\left(x\right),\;\;f\left(x,y\left(x\right)\right)=\overset{\infty }{\underset{j=0}{\sum }}A_{j}, \end{array}$$

where *A*_*j*_ are Adomian's polynomials which can be constructed for various classes of nonlinear functions with the formula given in [13] as

(6) $$\begin{array}{c} A_{n}=\frac{1}{n!}\frac{d^{n}}{d\lambda^{n}}{\left[f\left(x,\overset{\infty }{\underset{k=0}{\sum }}y_{k}\lambda^{k}\right)\right]}_{\lambda =0},\;n=0,1,2,\ldots ,. \end{array}$$

Substituting the series (5) into (4), we obtain

(7) $$\begin{array}{c} \overset{\infty }{\underset{j=0}{\sum }}y_{j}\left(x\right)=y\left(0\right)+L^{-1}\left[\overset{\infty }{\underset{j=0}{\sum }}A_{j}\right]. \end{array}$$

Upon matching both sides of (7), the ADM is given by

(8) $$\begin{array}{c} y_{0}\left(x,\eta \right)=\eta ,\;\;y_{j}\left(x,\eta \right)=L^{-1}\left[A_{j-1}\right],\;j\geq 1, \end{array}$$

where *y*(0) = *η* ≠ 0 is unknown constant to be determined. Having determined the components *y*_*n*_(*x*, *η*), the series solution of *y*(*x*) follows immediately with the undetermined *η*. Hence, the *n*-term truncated series solution is given as

(9) $$\begin{array}{c} \phi_{n}\left(x,\eta \right)=\overset{n}{\underset{j=0}{\sum }}y_{j}\left(x,\eta \right). \end{array}$$

It should be noted that the approximate solution *ϕ*_*n*_(*x*, *η*) depends on the unknown constant *η*. This constant *η* can be obtained approximately by imposing the boundary condition at *x* = 1 on *ϕ*_*n*_(*x*, *η*), which leads to a sequence of transcendental equations *aϕ*_*n*_(1, *η*) + *bϕ*_*n*_′(1, *η*) = *cn* = 1,2, 3,…. For example, consider

(10) $$\begin{array}{c} y^{\prime \prime }\left(x\right)=-e^{y(x)},\;\;y\left(0\right)=y\left(1\right)=0. \end{array}$$

According to the ADM (8), with *y*_0_(*x*, *η*) = *ηx*, we obtain the components as

(11) y0(x,η)=ηx,y1(x,η)=(1η2−eηxη2+xη),y2(x,η)=(−54η4+eηxη4+e2ηx4η4−x2η3−eηxxη3),⋮

Consequently, the *n*-term approximate series solution can be obtained as

(12) $$\begin{array}{c} \phi_{n}\left(x,\eta \right)=\overset{n}{\underset{j=0}{\sum }}y_{j}\left(x,\eta \right). \end{array}$$

By imposing the boundary condition at *x* = 1 on *ϕ*_*n*_(*x*, *η*), we obtain a sequence of transcendental equations *ϕ*_*n*_(1, *η*) = 0, *n* = 1,2,… as follows

(13) ϕ1(1,η)≡η+(1η2−eηη2+1η)=0,ϕ2(1,η)≡η+(1η2−eηη2+1η)     +(−54η4+eηη4+e2η4η4−12η3−eηη3)=0,    ⋮

However, solving such transcendental equations for *η* requires additional computational work, and *η* may not be uniquely determined. This may be the major disadvantage of the ADM for solving two-point boundary value problems.

## 2. The Proposed Recursive Scheme

In this section, we propose a new efficient recursive scheme based on the ADM for solving nonlinear SBVPs (1). To overcome the singular behavior at *x* = 0, we rewrite SBVPs (1) in operator form as

(14) $$\begin{array}{c} ℒy\left(x\right)=x^{\alpha }f\left(x,y\left(x\right)\right),\;0<x<1 \end{array}$$

subject to the boundary conditions

(15) $$\begin{array}{c} y^{\prime }\left(0\right)=0,\;\;ay\left(1\right)+by^{\prime }\left(1\right)=c, \end{array}$$

where *ℒ* = *d*/*dx*(*x* ^*α*^ *d*/*dx*) is a second-order linear singular differential operator. Twofold integral operator *ℒ* ^−1^[·] regarded as the inverse operator of *ℒ* is proposed as

(16) $$\begin{array}{c} {ℒ}^{-1}\left[\cdot \right]=\overset{1}{\underset{x}{\int }}s^{-\alpha }\left[\overset{s}{\underset{0}{\int }}\left[\cdot \right]dx\right]ds. \end{array}$$

In order to establish the new efficient recursive scheme, we operate *ℒ* ^−1^[·] on left hand side of (14) and impose the boundary condition *y*′(0) = 0; we obtain

(17) ℒ−1[(xαy′(x))′] =∫x1s−α[∫0s(xαy′(x))′dx]ds, =∫x1s−α[∫0s(xαy′′(x)+αxα−1y′(x))dx]ds, =∫x1s−α[sαy′(s)−∫0sαxα−1y′(x)dx       +∫0sαxα−1y′(x)dx]ds, =∫x1y′(s)ds=y(1)−y(x).

Thus we have

(18) $$\begin{array}{c} {ℒ}^{-1}\left[{\left(x^{\alpha }y^{\prime }\left(x\right)\right)}^{\prime }\right]=c_{0}-y\left(x\right), \end{array}$$

where *c*_0_ = *y*(1) ≠ 0 is unknown constant to be determined.

We now operate *ℒ* ^−1^[·] on both sides of (14) and use (18); we get

(19) $$\begin{array}{c} y\left(x\right)=c_{0}-\overset{1}{\underset{x}{\int }}s^{-\alpha }\left[\overset{s}{\underset{0}{\int }}x^{\alpha }f\left(x,y\left(x\right)\right)dx\right]ds. \end{array}$$

To eliminate unknown constant *c*_0_, we impose the boundary condition *ay*(1) + *by*′(1) = *c* on (19) which leads to

(20) $$\begin{array}{c} c_{0}=\frac{c}{a}-\frac{b}{a}\overset{1}{\underset{0}{\int }}x^{\alpha }f\left(x,y\left(x\right)\right)dx. \end{array}$$

Substituting the value of *c*_0_ into (19), we obtain

(21) y(x)=ca−ba∫01xαf(x,y(x))dx −∫x1s−α[∫0sxαf(x,y(x))dx]ds.

Note that (21) does not involve any unknown constants to be determined.

We now decompose the solution *y*(*x*) by the series as

(22) $$\begin{array}{c} y\left(x\right)=\overset{\infty }{\underset{j=0}{\sum }}y_{j}\left(x\right),\;\;f\left(x,y\left(x\right)\right)=\overset{\infty }{\underset{j=0}{\sum }}A_{j}, \end{array}$$

where *A*_*n*_ are the Adomian's polynomials [13]. In 2010, Duan [25, 26] reported several new efficient algorithms for rapid computer generation of the Adomian polynomials. Recently, El-Kalla [27] suggested another programmable formula for Adomian polynomials:

(23) $$\begin{array}{c} A_{n}=f\left(x,\psi_{n}\right)-\overset{n-1}{\underset{j=0}{\sum }}A_{j}\;\text{or}\;\;f\left(x,\psi_{n}\right)=\overset{n}{\underset{j=0}{\sum }}A_{j}, \end{array}$$

where *ψ*_*n*_ = ∑_*j*=0_^*n*^ *y*_*j*_ is partial sum of the series solution ∑_*j*=0_^*∞*^ *y*_*j*_.

Substituting the series (22) into (21), we obtain

(24) ∑j=0∞yj(x)=ca−ba∫01xα[∑j=0∞Aj]dx −∫x1s−α[∫0sxα∑j=0∞Ajdx]ds.

Comparing both sides of (24), the proposed scheme is given by the following recursive scheme:

(25) y0(x)=ca,yj(x)  =−ba∫01xαAj−1dx−∫x1s−α[∫0sxαAj−1dx]ds,  j≥1.

The recursive scheme (25) provides complete determination of solution components *y*_*j*_(*x*) of the solution *y*(*x*). Hence, the truncated *n*-term series solution can be obtained as

(26) $$\begin{array}{c} \psi_{n}\left(x\right)=\overset{n}{\underset{j=0}{\sum }}y_{j}\left(x\right). \end{array}$$

Unlike ADM or MADM, the proposed recursive scheme (25) does not require any computation of unknown constants.

## 3. Convergence Analysis

In this section, we will give the sufficient condition that guarantees existence of a unique solution of (28) in Theorem 1. Then we discuss the convergence analysis in Theorem 2 and the error analysis in Theorem 3 of the proposed scheme (25) for SBVPs (1). Note that many researcher [28–31] have also established the convergence of the ADM for solving differential as well as integral equations. Let *𝕏* = *C*[0,1] be a Banach space with the norm

(27) $$\begin{array}{c} ||y||=\underset{x\in \left[0,1\right]}{\max}\left|y\left(x\right)\right|,\;y\in 𝕏. \end{array}$$

Note that (21) can be written in operator form as

(28) $$\begin{array}{c} y=𝒩\left(y\right), \end{array}$$

where *𝒩*(*y*) is given by

(29) 𝒩(y)=ca−ba∫01xαf(x,y)dx −∫x1s−α[∫0sxαf(x,y)dx]ds.

**Theorem 1**

Let *𝕏* be a Banach space with the norm given by (27). Assuming that the nonlinear function *f*(*x*, *y*) satisfies the Lipschitz condition; that is, |*f*(*x*, *y*) − *f*(*x*, y*)|≤*L* | *y* − y*|. Further, let *δ* be a constant defined as

(30) $$\begin{array}{c} \delta ∶=\frac{L\left(a+2b\right)}{2a\left(1+\alpha \right)}. \end{array}$$

If *δ* < 1, then (28) has a unique solution in *𝕏*.

**Proof**

For any *y*, y* ∈ *𝕏*, we have

(31) ||𝒩(y)−𝒩(y∗)|| =max⁡x∈[0,1]|ba∫01xα[f(x,y)−f(x,y∗)]dx      +∫x1s−α[∫0sxα[f(x,y)−f(x,y∗)]dx]ds|, ≤max⁡x∈[0,1]|f(x,y)−f(x,y∗)|  ×max⁡x∈[0,1]|ba∫01xαdx+∫x1s−α[∫0sxαdx]ds|.

Using Lipschitz continuity of *f*, we obtain

(32) $$\begin{array}{c} ||𝒩\left(y\right)-𝒩\left(y^{*}\right)||\leq \frac{L\left(a+2b\right)}{2a\left(1+\alpha \right)}\underset{x\in \left[0,1\right]}{\max}\left|y-y^{*}\right|=\delta ||y-y^{*}||. \end{array}$$

Hence, we have

(33) $$\begin{array}{c} ||𝒩\left(y\right)-𝒩\left(y^{*}\right)||\leq \delta ||y-y^{*}||. \end{array}$$

If *δ* < 1, then *𝒩* : *𝕏* → *𝕏* is contraction mapping and hence by the Banach contraction mapping theorem, (28) has a unique solution in *𝕏*.

We now prove the convergence of the proposed scheme (25). Let {*ψ*_*n*_ = ∑_*j*=0_^*n*^ *y*_*j*_} be a sequence of partial sums of the series solution ∑_*j*=0_^*∞*^ *y*_*j*_. Using (25) and (26), we have

(34) ψn=∑j=0nyj=y0+∑j=1nyj=ca −∑j=1n[ba∫01xαAj−1dx+∫x1s−α[∫0sxαAj−1dx]ds]=ca−ba∫01xα∑j=1nAj−1dx −∫x1s−α[∫0sxα∑j=1nAj−1dx]ds.

Using (23) in (34), it follows that

(35) ψn=ca−ba∫01xαf(x,ψn−1)dx −∫x1s−α[∫0sxαf(x,ψn−1)dx]ds,

which is equivalent to the following operator equation:

(36) $$\begin{array}{c} \psi_{n}=𝒩\left(\psi_{n-1}\right),\;n=1,2,\ldots \text{.} \end{array}$$

Note that the formulation (36) is used to prove Theorems 2 and 3.

**Theorem 2**

Let *𝒩*(*y*) be the nonlinear operator defined by (28) as contractive; that is,

(37) ||𝒩(y)−𝒩(y∗)||≤δ||y−y∗||, ∀y,y∗∈𝕏,   with δ<1,  ||y1||<∞.

Then the sequence {*ψ*_*n*_} of partial sums defined by (26) converges to the exact solution *y*.

**Proof**

Using the relation (36) and the estimate (33), we have

(38) ||ψm+1−ψm|| =||𝒩(ψm)−𝒩(ψm−1)||≤δ||ψm−ψm−1||.

Thus we have

(39) ||ψm+1−ψm||≤δ||ψm−ψm−1||≤δ2||ψm−1−ψm−2||≤⋯≤δm||ψ1−ψ0||.

We now show that the sequence {*ψ*_*n*_} is convergent. Now for all *n*, *m* ∈ *ℕ*, with *n* ≥ *m*, we consider

(40) ||ψn−ψm|| =||(ψn−ψn−1)+(ψn−1−ψn−2)+⋯+(ψm+1−ψm)|| ≤||ψn−ψn−1||+||ψn−1−ψn−2||+⋯+||ψm+1−ψm|| ≤[δn−1+δn−2+⋯+δm]||ψ1−ψ0|| =δm[1+δ+δ2+⋯+δn−m−1]||ψ1−ψ0|| =δm(1−δn−m1−δ)||y1||.

Since 0 < *δ* < 1, (1 − *δ* ^*n*−*m*^) < 1, and ||*y*_1_|| < *∞*, it follows

(41) $$\begin{array}{c} ||\psi_{n}-\psi_{m}||\leq \frac{\delta^{m}}{1-\delta }||y_{1}||. \end{array}$$

Taking limit as *m* → *∞*, we obtain

(42) $$\begin{array}{c} ||\psi_{n}-\psi_{m}||⟶0. \end{array}$$

Hence {*ψ*_*n*_} is cauchy sequence in *𝕏*. Hence there exits *ψ* in *𝕏* such that lim⁡_*n*→*∞*_ *ψ*_*n*_ = *ψ*. Note that *ψ* is the exact solution of (28) as

(43) $$\begin{array}{c} y=\overset{\infty }{\underset{j=0}{\sum }}y_{j}=\underset{n\to \infty }{\lim}\psi_{n}=\psi . \end{array}$$

This completes the proof.

**Theorem 3**

Let *y*(*x*) be the exact solution of the operator equation (28). Let *ψ*_*m*_(*x*) be the sequence of partial sums of series solution defined by (26). Then there holds

(44) $$\begin{array}{c} \underset{x\in \left[0,1\right]}{\max}\left|y\left(x\right)-\overset{m}{\underset{j=0}{\sum }}y_{j}\left(x\right)\right|\leq \frac{M\delta^{m+1}}{L\left(1-\delta \right)}, \end{array}$$

where *M* = max⁡_*x*∈[0,1]_ | *f*(*x*, *y*_0_)|.

**Proof**

For any *n* ≥ *m* and using the estimate (41), we have

(45) $$\begin{array}{c} ||\psi_{n}-\psi_{m}||\leq \frac{\delta^{m}}{1-\delta }||y_{1}||. \end{array}$$

Since lim⁡_*n*→*∞*_ *ψ*_*n*_ = *y*, fixing *m* and letting *n* → *∞* in above estimate, we obtain

(46) $$\begin{array}{c} ||y-\psi_{m}||\leq \frac{\delta^{m}}{1-\delta }\underset{x\in \left[0,1\right]}{\max}\left|y_{1}\left(x\right)\right|. \end{array}$$

Since, we have

(47) y1(x)=−ba∫01xαA0dx−∫x1s−α[∫0sxαA0dx]ds,               A0=f(x,y0),

so

(48) max⁡x∈[0,1]|y1(x)| =max⁡x∈[0,1]|ba∫01xαA0dx+∫x1s−α[∫0sxαA0dx]ds| ≤(a+2b)2a(1+α)max⁡x∈[0,1]|f(x,u0)|.

Combining the estimates (46) and (48) and using *δ*/*L* = (*a* + 2*b*)/(2*a*(1 + *α*)), we obtain

(49) $$\begin{array}{c} \underset{x\in [0,1]}{\max}\left|y\left(x\right)-\overset{m}{\underset{j=0}{\sum }}y_{j}\left(x\right)\right|\leq \frac{M\delta^{m+1}}{L\left(1-\delta \right)}. \end{array}$$

This completes the proof.

## 4. Numerical Examples

In this section, we demonstrate the accuracy and reliability of the proposed scheme (25) by implementing it to four SBVPs, arising various physical models. All the numerical results obtained by the proposed (25) are compared with the results obtained by various numerical methods.

**Example 1**

Consider the following nonlinear SBVPs:

(50) $$\begin{array}{c} {\left(x^{\alpha }y^{\prime }\left(x\right)\right)}^{\prime }=x^{\alpha }\left(\frac{N_{1}y\left(x\right)}{y\left(x\right)+K_{1}}\right),\;0<x<1,\\ y^{\prime }\left(0\right)=0,\;\;5y\left(1\right)+y^{\prime }\left(1\right)=5, \end{array}$$

where *N*_1_ and *K*_1_ are positive constants involving the reaction rate and Michaelis constants. Thus we take *N*_1_ = 0.76129 and *K*_1_ = 0.03119 as used in [1, 3, 5].

According to the proposed method (25) with *a* = 5, *b* = 1, and *c* = 5. Consequently, we have the following recursive scheme:

(51) y0(x)=1,yj(x) =−15∫01xαAj−1dx−∫x1s−α[∫0sxαAj−1dx]ds, j≥1.

Using the formula (6), Adomian's polynomials for *f*(*y*(*x*)) = 0.76129*y*(*x*)/(*y*(*x*) + 0.03119) with *y*_0_ = 1 are obtained as

(52) A0=0.738264,A1=0.02233y1(x),A2=−0.0216546y12(x)+0.02233y2(x),A3=0.0209996y13(x)−0.0433091y1(x)y2(x)   +0.02233y3(x) ⋮

*Case (α* = 1*).* Using (51) and (52), the components *y*_*j*_(*x*) of the solution *y*(*x*) are obtained as

(53) y0(x)=1,y1(x)=−0.25839224+0.18456588x2,y2(x)=0.00155580−0.00144247x2+0.00025758x4,y3(x)=0.00030986−0.0003527x2+0.0001270x4    −0.0000203x6   ⋮

The 6-term approximate series solution *ψ*_6_(*x*) = ∑_*j*=0_^6^ *y*_*j*_(*x*) is given by

(54) ψ6(x)=0.743551+0.182662x2+0.0004499x4 −0.0000453x6+5.181586×10−6x8 −5.061711×10−7x10+2.801167×10−8x12.

*Case (α* = 2*).* In a similar manner, we obtain the components *y*_*j*_(*x*) of solution *y*(*x*) as

(55) y0(x)=1,y1(x)=−0.17226149+0.12304392x2,y2(x)=0.0006502−0.0006410x2+0.0001373x4,y3(x)=0.0000822−0.0001046x2+0.000045x4    −7.732815×10−6x6   ⋮

Hence, we obtain the 6-term approximate series solution *ψ*_6_(*x*) = ∑_*j*=0_^6^ *y*_*j*_ as

(56) ψ6(x)=0.828483+0.122278x2+0.000196x4 −0.000013x6+1.013102×10−6x8 −7.367823×10−8x10+3.366698×10−9x12.

*Case (α* = 3*).* The components *y*_*j*_(*x*) of the solution *y*(*x*) are obtained as

(57) y0(x)=1,y1(x)=−0.1291961+0.0922829x2,y2(x)=0.000350−0.000360x2+0.0000858x4,y3(x)=0.0000321−0.0000442x2+0.000021x4    −3.802000×10−6x6   ⋮

The series solution is given as follows:

(58) ψ6(x)=0.87119+0.0918721x2+0.0001116x4 −5.600223×10−6x6+3.279844×10−7x8 −1.8874803×10−8x10+7.358761×10−10x12.

Comparison of the numerical results obtained by the proposed method (25), the VIM used in [5], and the CSM used in [1] are shown in Tables 1, 2, and 3. These tables show that the numerical results obtained by the proposed scheme (25) are comparable with those in [1, 5]. In addition, we have also presented numerical results for *α* = 0,4, 5 in Tables 1 and 3.

**Example 2**

Consider the following nonlinear SBVPs [9]:

(59) $$\begin{array}{c} {\left(x^{2}y^{\prime }\left(x\right)\right)}^{\prime }=x^{2}y^{5}\left(x\right),\;0<x<1,\\ y^{\prime }\left(0\right)=0,\;\;y\left(1\right)=\frac{\sqrt{3}}{2}. \end{array}$$

The analytical solution is $y(x)=\sqrt{3/\left(3+x^{2}\right)}$.

According to the proposed method (25) with *α* = 2, *a* = 1, *b* = 0, and $c=\sqrt{3}/2$. Consequently, we have the following recursive scheme as

(60) y0(x)=32,yj(x) =−01∫01x2Aj−1dx−∫x1s−2[∫0sx2Aj−1dx]ds, j≥1.

Similar to the previous example, Adomian's polynomials for *f*(*y*(*x*)) = *y* ^5^(*x*) are given as

(61) A0=y05(x),A1=5y04y1(x),A2=10y03(x)y12(x)+5y04(x)y2(x),A3=10y02(x)y13(x)+20y03(x)y1(x)y2(x)  +5y04(x)y3(x) ⋮

Using (60) and (61), we obtain the components *y*_*j*_(*x*) of the solution *y*(*x*) as

(62) y0(x)=0.8660254,y1(x)=0.0811899−0.0811899x2,y2(x)=0.0266404−0.0380578x2+0.0114173x4,y3(x)=0.0117741−0.0196235x2+0.00963337x4  −0.00178396x6⋮

Hence, we obtain approximate series solution as

(63) ψ6(x)=0.996812−0.159895x2+0.0354173x4 −0.00743284x6+0.00126204x8 −0.000146626x10+8.48888×10−6x12.

For quantitative comparison, we now define the maximum absolute error by

(64) $$\begin{array}{c} E_{n}=\underset{0<x\leq 1}{\max}\left|\psi_{n}\left(x\right)-y\left(x\right)\right|. \end{array}$$

Here *ψ*_*n*_(*x*) is the *n*-term approximate series solution obtained by the proposed method (25) and *y*(*x*) is the analytical solution.  Table 4 shows a comparison between the numerical results obtained by the proposed method (25) and the FDM used in [9]. The numerical results obtained by the proposed method (25) show good agreement with those in [9].

**Example 3**

Consider the following nonlinear SBVPs [3]:

(65) $$\begin{array}{c} {\left(xy^{\prime }\left(x\right)\right)}^{\prime }=-xe^{y(x)},\;0<x<1,\\ y^{\prime }\left(0\right)=0,\;\;y\left(1\right)=0. \end{array}$$

The analytical solution is *y*(*x*) = 2ln⁡((*C* + 1)/(*Cx* ^2^ + 1)), where $C=3-2\sqrt{2}$.

According to the proposed method (25) with *α* = 1, *a* = 1, *b* = 0, and *c* = 0, we have the following recursive scheme:

(66) y0(x)=0,yj(x) =01∫01xAj−1dx+∫x1s−1[∫0sxAj−1dx]ds, j≥1.

Proceeding as before, Adomian's polynomials for *f*(*y*(*x*)) = *e* ^*y*(*x*)^ with *y*_0_(*x*) = 0 are given as

(67) A0=1,A1=y1(x),A2=y12(x)2+y2(x),A3=y13(x)6+y1(x)y2(x)+y3(x)⋮

Making use of (66) and (67), we can calculate the components *y*_*j*_(*x*) of solution *y*(*x*) as

(68) y0(x)=0,y1(x)=0.25−0.25x2,y2(x)=0.046875−0.0625x2+0.015625x4,y3(x)=0.0130208−0.0195313x2+0.0078125x4 −0.00130208x6⋮

Hence we obtain approximate series solution as

(69) ψ6(x)=0.316294−0.342438x2+0.0289497x4 −0.00310771x6+0.000328064x8 −0.000027465x10+1.2715657×10−6x12.

Table 5 shows a comparison of the numerical results obtained by the proposed method (25), the FDM used in [9], and the BSM used in [2]. Once again, the approximate series solutions using proposed method (25) are comparable with the results reported earlier in the literature [2, 9].

**Example 4**

Consider the following nonlinear SBVPs [8]:

(70) $$\begin{array}{c} {\left(x^{2}y^{\prime }\left(x\right)\right)}^{\prime }=-x^{2}e^{-y(x)},\;0<x<1,\\ y^{\prime }\left(0\right)=0,\;\;2y\left(1\right)+y^{\prime }\left(1\right)=0. \end{array}$$

According to the proposed method (25) with *α* = 2, *a* = 2, *b* = 1, and *c* = 0, we have the following scheme:

(71) y0(x)=0,yj(x) =12∫01x2Aj−1dx+∫x1s−2[∫0sx2Aj−1dx]ds, j≥1.

Adomian's polynomials, for *f*(*y*(*x*)) = *e* ^−*y*(*x*)^ with *y*_0_(*x*) = 0 are given as

(72) A0=1,A1=−y1(x),A2=y12(x)2−y2(x),A3=−y13(x)6+y1(x)y2(x)−y3(x) ⋮

Making use of (71) and (72), we obtain the component *y*_*n*_(*x*) as follows:

(73) y0(x)=0,y1(x)=0.333333−0.166667x2,y2(x)=−0.0861111+0.0555556x2−0.00833333x4,y3(x)=0.032672−0.0236111x2+0.00555556x4 −0.000529101x6⋮

Hence, the 6-term approximate series solution is given by

(74) ψ6(x)=0.270736−0.127835x2−0.00461112x4 −0.00030756x6+3.17554×10−6x8 −3.22937×10−6x10+2.18422×10−7x12 −1.74996×10−8x14.

Table 6 presents a comparison of the approximate solutions obtained by the proposed method (25), the FDM used in [32], and tangent chord technique used in [8]. We again obtain the numerical results using the proposed scheme (25) which are comparable with those in [8, 32].

## 5. Conclusions

- In this work, the simplicity, efficiency, and reliability of the proposed method (25) have been tested by solving four nonlinear SBVPs that arise in physiology.
- The accuracy of the numerical results indicates that the proposed method is well suited for the solution of SBVPs. It has also been shown that only 6 terms are sufficient to obtain comparable solution with those in [2, 9].
- Unlike ADM or MDAM, the proposed method (25) does not require any computation of unknown constants. In fact, it provides a direct scheme to obtain approximations to the solution.
- Unlike the finite difference and the cubic spline methods, the proposed method does not require any linearization and discretization of the variables.
- Uniqueness of the solution of (28) has been discussed in Theorem 1. The convergence analysis and the error estimation of the proposed scheme (25) have also been established in Theorems 2 and 3.

## Figures and Tables

**Table 1:** Numerical results for *α* = 0,1 of Example 1.

*x*	*ψ*_6_, *α* = 0	Wazwaz [5]	*ψ*_6_, *α* = 1	Wazwaz [5]
0.0	0.4950040961	0.4952605157	0.7435513167	0.7435531556
0.2	0.5093399331	0.5095882966	0.7508585027	0.7508602912
0.4	0.5523907867	0.5526180550	0.7727885297	0.7727901915
0.6	0.6242765760	0.6244730118	0.8093658234	0.8093673219
0.8	0.7251677449	0.7253051125	0.8606280633	0.8606293419
1.0	0.8552538780	0.8552148933	0.9266223859	0.9266231833

**Table 2:** Numerical results for *α* = 2 of Example 1.

*x*	*ψ*_4_	*ψ*_6_	Waswaz [5]	Kanth and Bhattacharya [1]
0.0	0.8284816685	0.8284832618	0.8284832761	0.8284832730
0.2	0.8333732324	0.8333747079	0.8333747193	0.8333747169
0.4	0.8480515962	0.8480527660	0.8480527701	0.8480527704
0.6	0.8725275171	0.8725283082	0.8725282997	0.8725283084
0.8	0.9068180902	0.9068185418	0.9068185095	0.9068185403
1.0	0.9509455924	0.9509457957	0.9509457539	0.9509457946

**Table 3:** Numerical results for *α* = 3,4, 5 of Example 1.

*x*	*ψ*_6_, *α* = 3	Waswaz [5]	*ψ*_6_, *α* = 4	*ψ*_6_, *α* = 5
0.0	0.8711896978	0.8711897615	0.8968770467	0.9140255488
0.2	0.8748647597	0.8748648229	0.8998199822	0.9164795274
0.4	0.8858920668	0.8858921290	0.9086501684	0.9238424346
0.6	0.9042778592	0.9042779179	0.9233716779	0.9361171460
0.8	0.9300321361	0.9300321834	0.9439910856	0.9533083270
1.0	0.9631681076	0.9631681318	0.9705171710	0.9754222526

**Table 4:** Comparison of the maximum absolute error of Example 2.

*n*	*E*_*n*_	*n*	Chawla et al. [9]
5	5.0992 × 10^−3^	16	3.64 × 10^−4^
10	9.2468 × 10^−5^	32	2.49 × 10^−5^
15	2.1193 × 10^−6^	64	1.60 × 10^−6^

**Table 5:** Comparison of the maximum absolute error of Example 3.

*n*	*E*_*n*_	*n*	Ça$\overset{˘}{\text{g}}$lar et al. [2]	*n*	Chawla et al. [9]
5	6.2129 × 10^−4^	20	3.1606 × 10^−5^	16	2.52 × 10^−3^
10	1.1318 × 10^−6^	40	7.8742 × 10^−6^	32	1.83 × 10^−4^
15	2.0760 × 10^−7^	60	3.5011 × 10^−6^	64	1.28 × 10^−5^

**Table 6:** Numerical results of Example 4.

*x*	*ψ*_6_	*ψ*_8_	*ψ*_10_	Pandey [32]	Duggan and Goodman [8]
0.0	0.270736276	0.270258951	0.270108917	—	0.270350067
0.2	0.265615467	0.265154126	0.265009271	0.264932820	0.265254341
0.4	0.250163329	0.249747091	0.249616772	0.249548183	0.249867127
0.6	0.224103659	0.223753983	0.223644912	0.223587710	0.223885976
0.8	0.186952558	0.186680714	0.186596204	0.186552018	0.186798950
1.0	0.137982462	0.137789790	0.137729980	0.137698751	0.137872638
